# Supplementary material for: μ₃-Oxo nucleophile formation enables efficient SN2 hydrolysis at the trinuclear metal center in inorganic pyrophosphatase
Source: Commun Chem. 2026 Apr 2;9:190. doi: 10.1038/s42004-026-01996-7 (PMC13219714; doi:10.1038/s42004-026-01996-7)
Supplement: Supplementary file 2 — Supplementary Information [file 42004_2026_1996_MOESM2_ESM.pdf]

μ_3-Oxo nucleophile formation enables efficient S_N2 hydrolysis at the trinuclear metal center in inorganic pyrophosphatase

Saki Maruoka[1,4], Yohei Kametani[2], Eisuke Magome[3], Hiroyuki Setoyama[3], Masahide Kawamoto[3], Masaki Horitani[4], Takamasa Teramoto[1], Yoshimitsu Kakuta[1], Yoshihito Shiota[2], Kazunari Yoshizawa[2,5], and Keiichi Watanabe[4,6] *

[1]Laboratory of Biophysical Chemistry, Department of Bioscience and Biotechnology, Faculty of Agriculture, Kyushu University, Fukuoka, Japan
[2]Institute for Materials Chemistry and Engineering and IRCCS, Kyushu University, Fukuoka, Japan
[3]SAGA Light Source, Tosu, Japan
[4]Department of Applied Biochemistry and Food Science, Saga University, Saga, Japan
[5]Fukui Institute for Fundamental Chemistry, Kyoto University, Kyoto, Japan
[6]Department of Data Science in Food Environment, Kyushu Nutrition Welfare University, Kitakyushu, Japan

SUPPLEMENTARY INFORMATION (SI)

Contents

1 Supplementary Figure 1 ………………..………………………..….. 2
2 Supplementary Figure 2 …………..…………………………….…. 3
3 Supplementary Figure 3 …………..…………………………….…. 4
4 Supplementary Figure 4 …………...…………………………….…. 5
5 Supplementary Figure 5 …………..…………………………….…. 6
6 Supplementary Figure 6 ……………...…………………………….…. 7
7 Supplementary Figure 7 ……………….……………………………….. 8
8 Supplementary Figure 8 ……………..…………………………….. 9
9 Supplementary Figure 9 ………………..……………………… 10
10 Supplementary Figure 10 …………..…………………………….. 11
11 Supplementary Figure 11 ………………..……………………... 12
12 Supplementary Table 1 ……………..…………………………….. 13
13 Supplementary Table 2 ……………...…………………………..… 14
14 Supplementary Table 3 ………………..…………………………..… 15

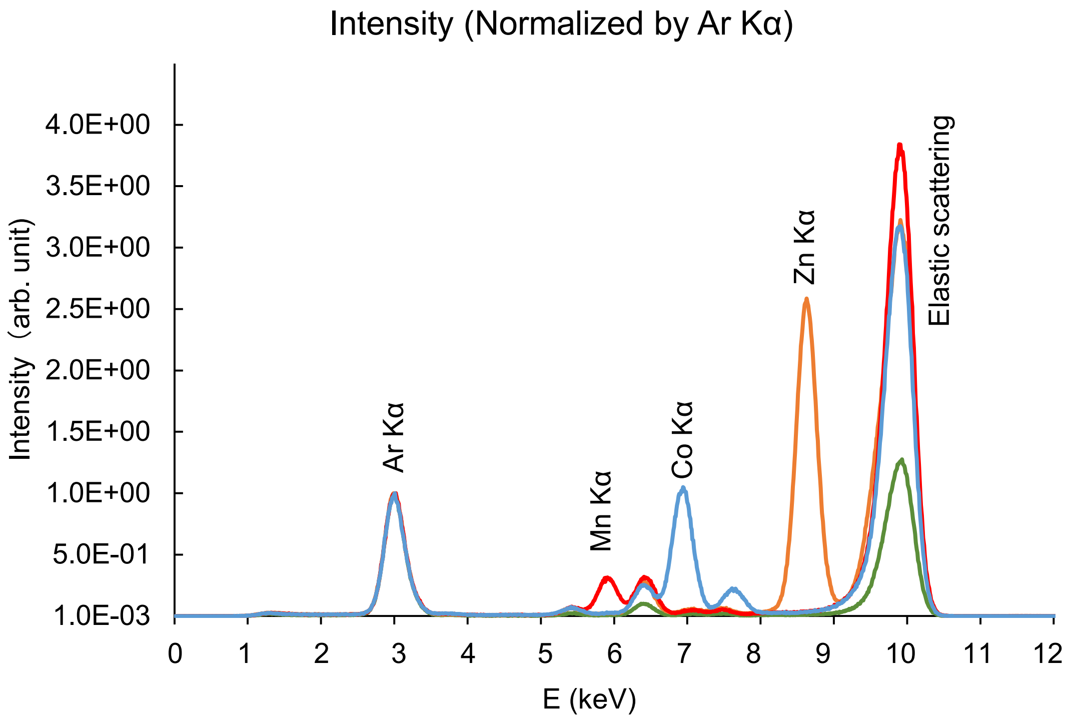

Supplementary Figure 1 | Metal-Kα emission measured by fluorescence x-ray detector. Kα emission spectra of 2 mM metal solutions as standard were measured. Zn, Mn, and Co are shown in orange, red, and light blue lines, respectively. Blank by PCR tube was shown in green line.

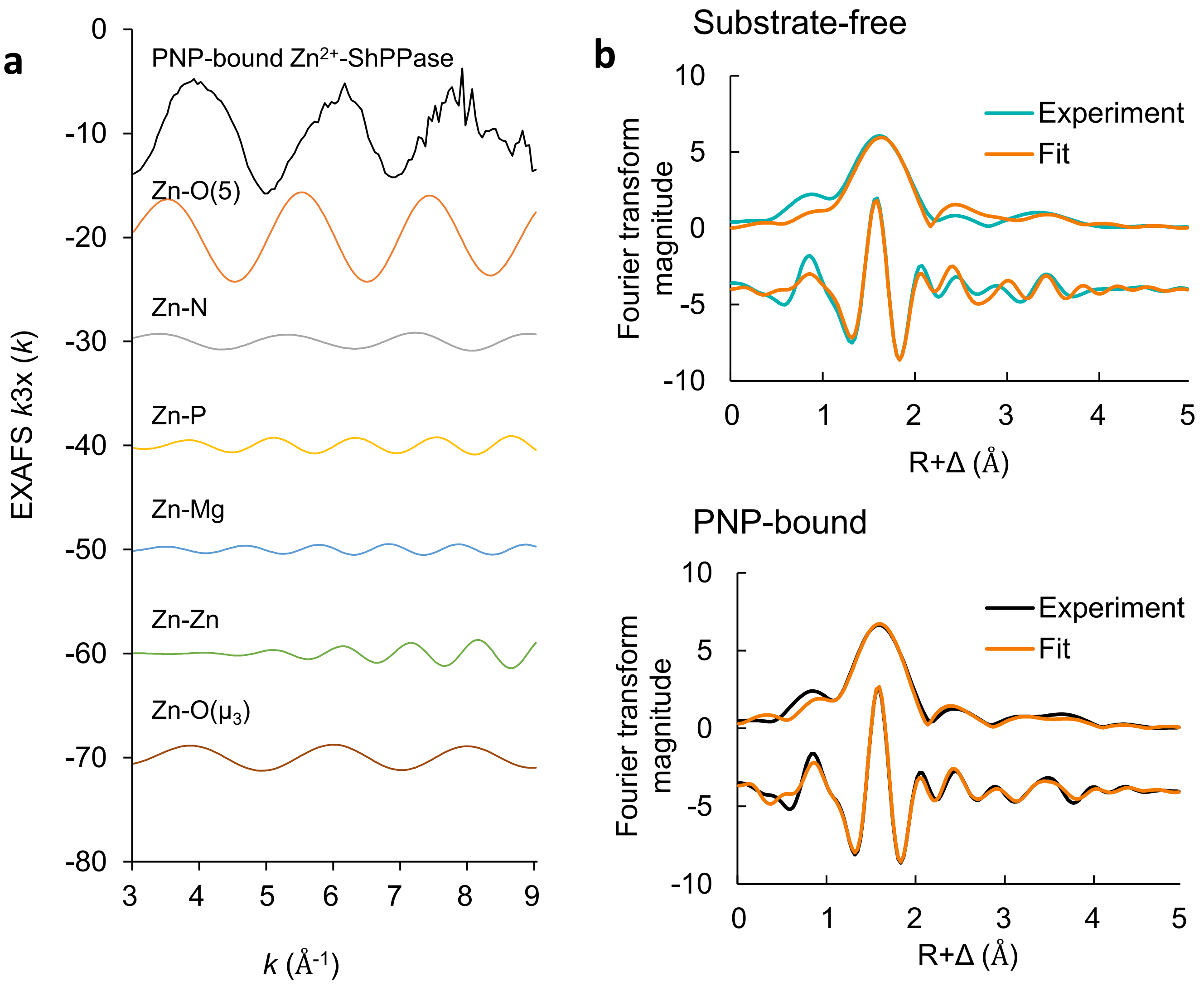

Supplementary Figure 2 | a, Comparison of EXAFS spectra between experimental and FEFF simulated spectra of the Zn-ShPPase. Scattering paths from FEFF include 4 oxygen atoms (orange line), a nitrogen atom (gray line), a phosphorus atom (yellow line), a magnesium atom (cyan), a zinc atom (green line), an oxygen atom derived from water which is coordinated with three metal cations (brown line). b, Fourier-transformed EXAFS spectra with best fit simulations. Upper trace: magnitude. Lower trace: imaginary component. Experimental data for the substrate-free (teal) and PNP-bound (black) forms; global fits (orange). Distances are shown as apparent R+Δ (phase-uncorrected radial distance).

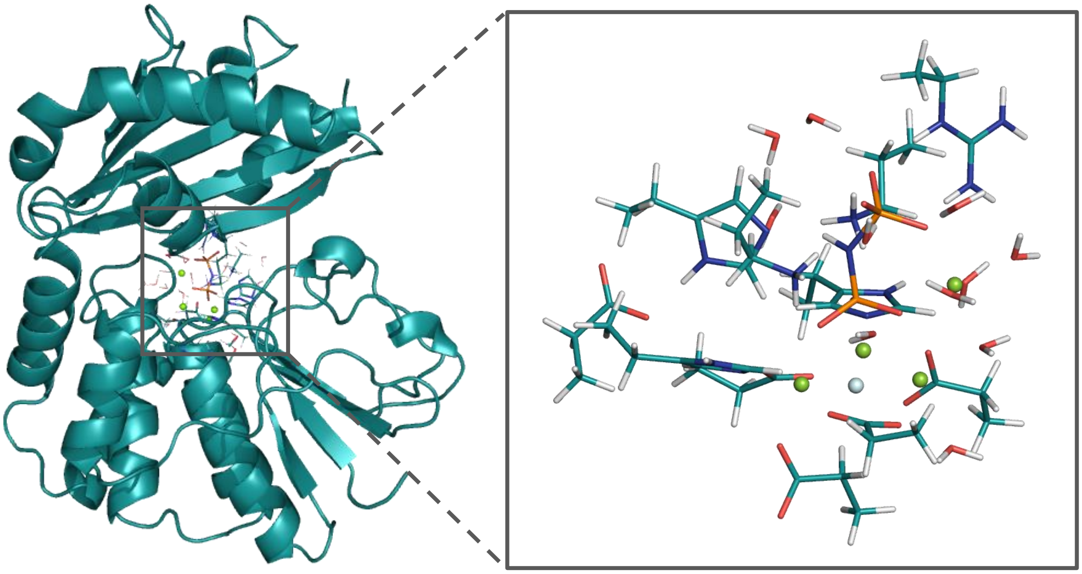

Supplementary Figure 3 | X-ray crystal structure (XSC) model of QM cluster models for the active site is depicted with the conventional licorice colors (C cyan, H white, O red, N blue, and P orange). Mg(II) ions and F are shown as green and light blue spheres, respectively.

X-ray crustal structure model of PNP-bound Mg^{2+}-ShPPase

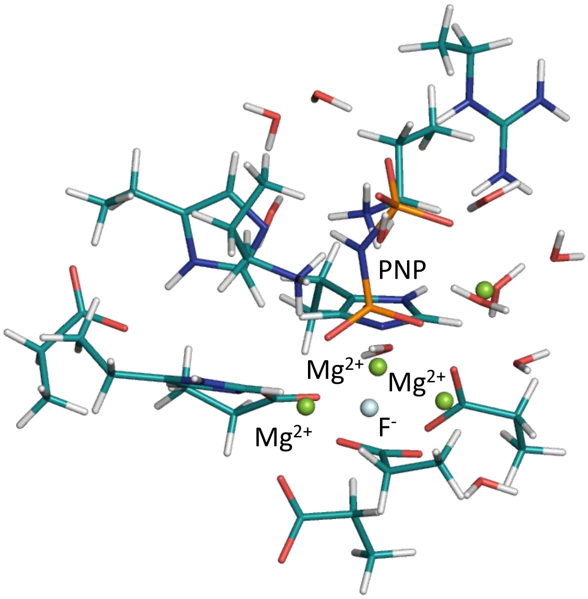

EXAFS model

a μ_3-hydroxide state

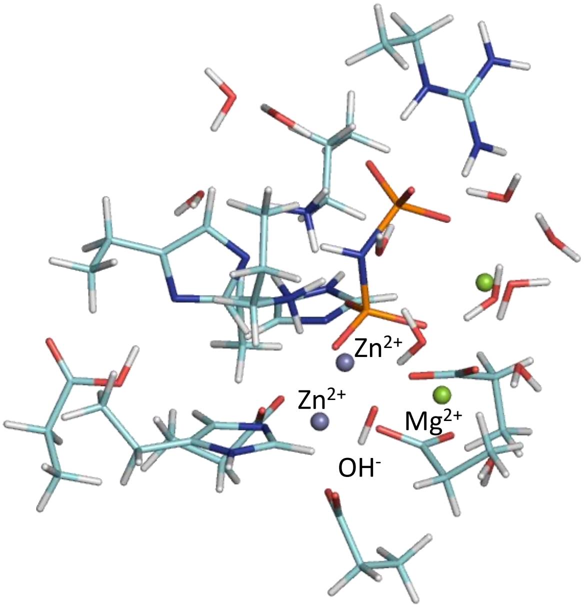

b μ_3-oxo state

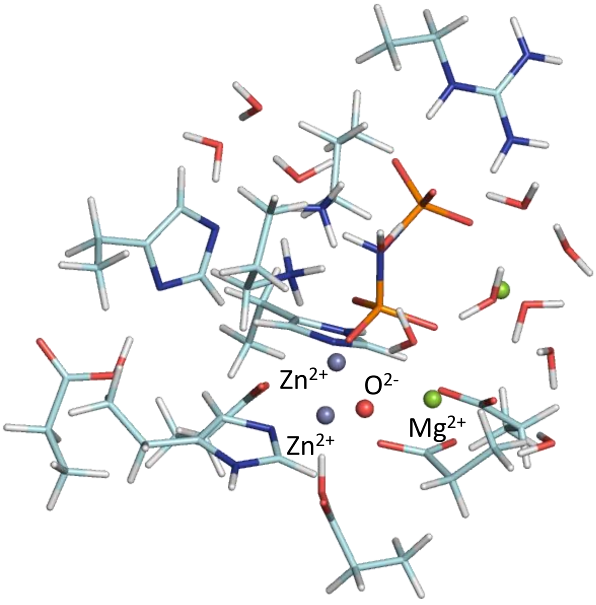

Supplementary Figure 4 | Optimized structures of X-ray crystal structure models and EXAFS models ((a) μ_3-hydroxide state, and (b) μ_3-oxo state). The Zn, Mg, O, N, P, and H atoms are colored slate purple, green, red, blue, orange, and white, respectively.

Asp14 rotation model with bound POP

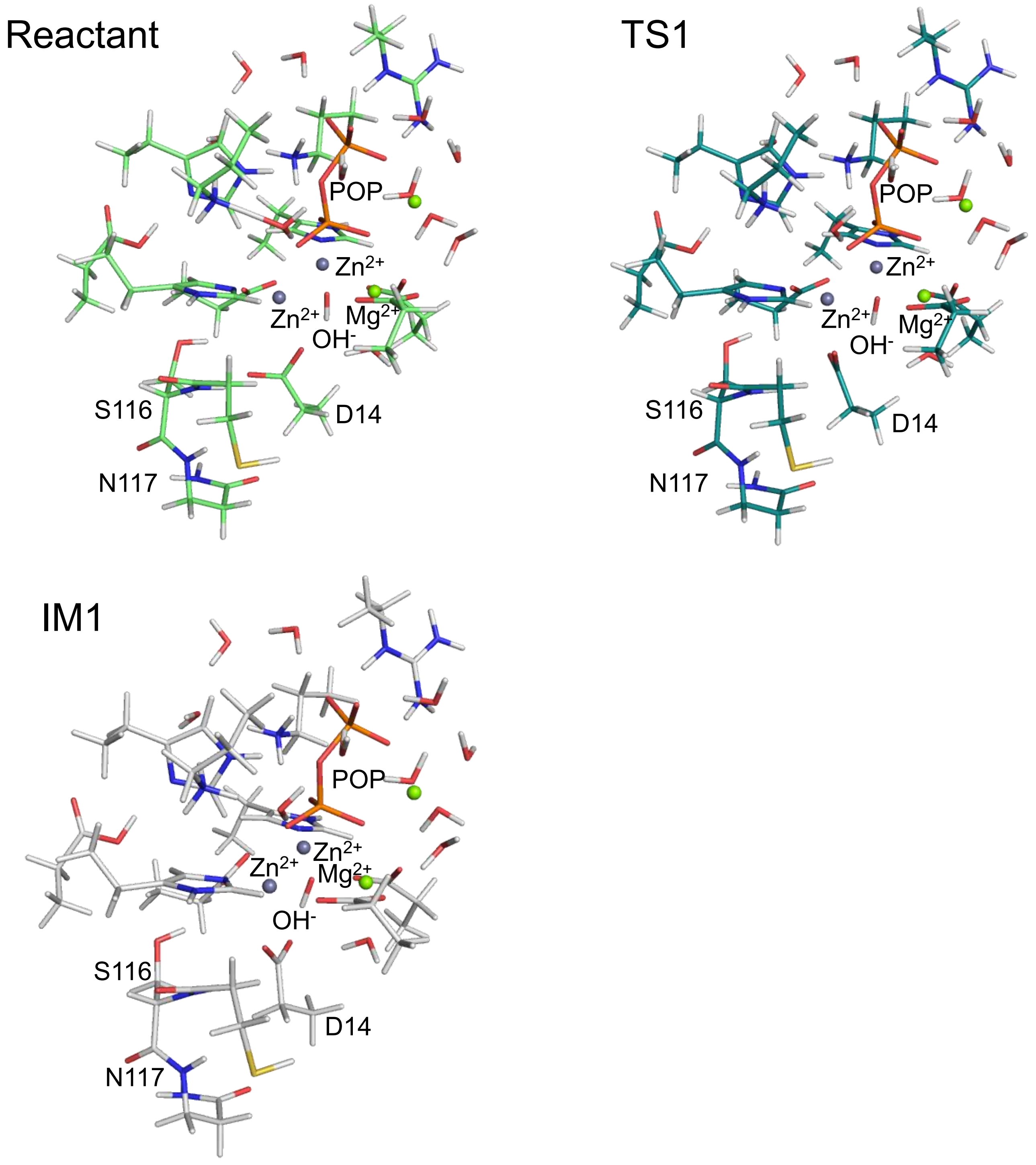

Supplementary Figure 5 | Optimized structures of Asp14 rotation models. The Zn, Mg, O, N, P, and H atoms are colored slate purple, green, red, blue, orange, and white, respectively.

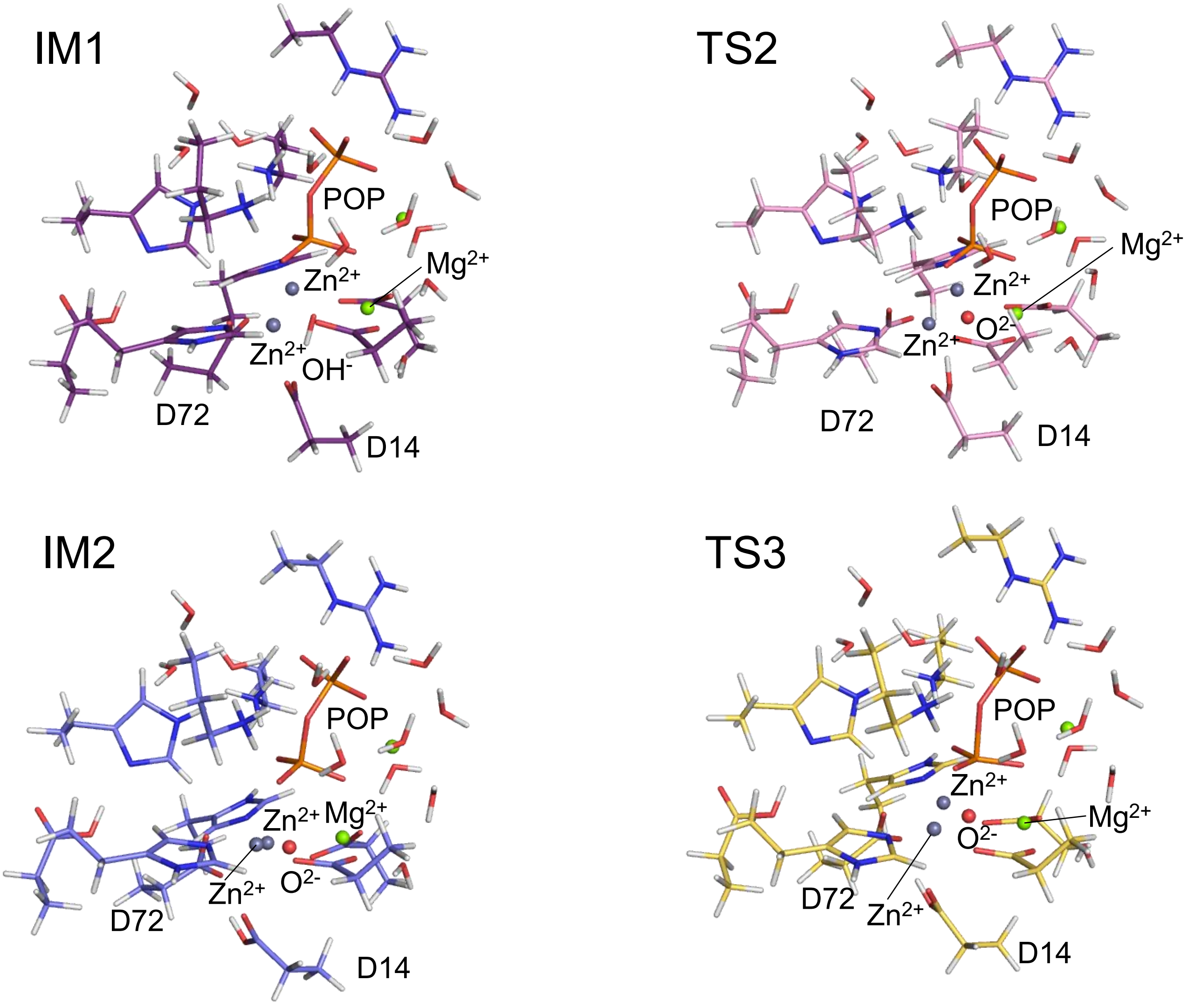

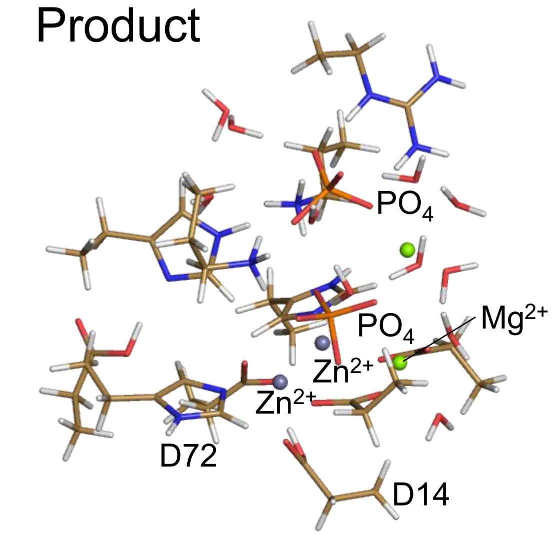

Supplementary Figure 6 | Optimized structures of μ$_3$-oxo formation and S_N2 reaction models with POP. The Zn, Mg, O, N, P, and H atoms are colored slate purple, green, red, blue, orange, and white, respectively.

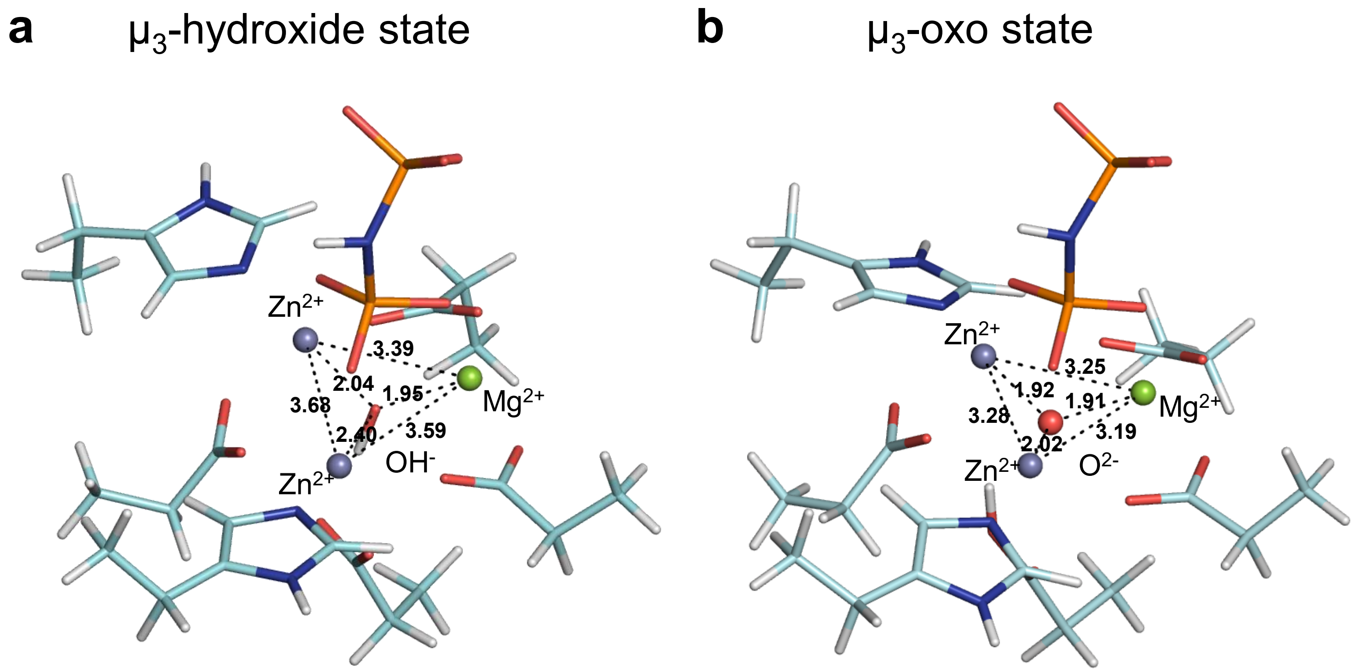

Supplementary Figure 7 | Optimized structures of EXAFS model. (a) μ_3-hydroxide state, and (b) μ_3-oxo state. Black dashes indicate coordination bonds. Interatomic distances are presented in Å. Only the active site near the tri-metal structure and substrate analog are shown, see Supplementary Figure 3 for EXAFS model with the whole active site. The Zn, Mg, O, N, P, and H atoms are colored slate purple, green, red, blue, orange, and white, respectively.

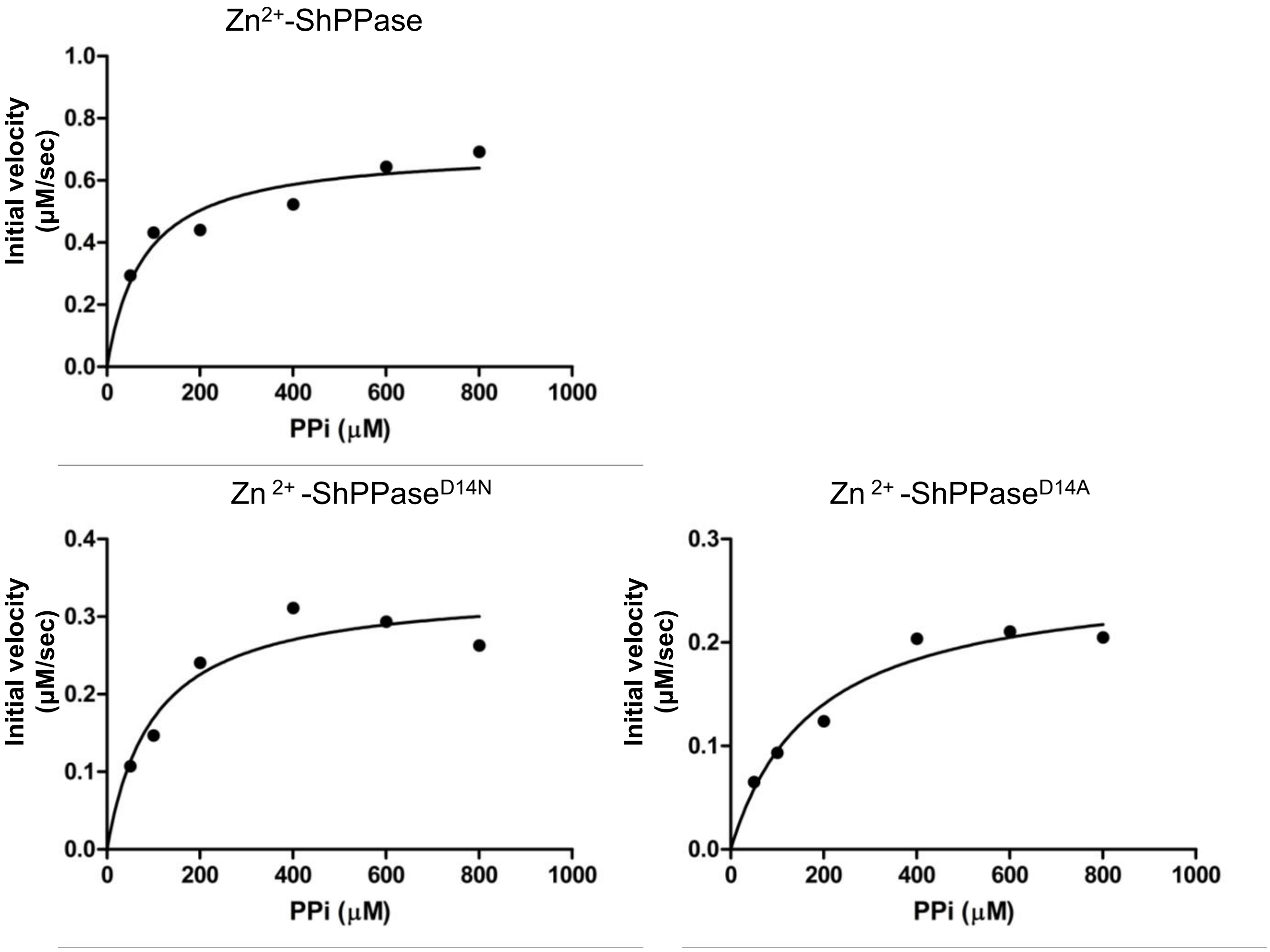

Supplementary Figure 8 | The kinetics of hydrolysis of the PPi substrate by Zn^{2+}-ShPPase, Zn^{2+}-$ShPPase^{D14N}$ and Zn^{2+}-$ShPPase^{D14A}$. Experimental data and fitted curves are shown in black dots and solid lines, respectively. The enzyme concentrations used in the assays were 2.9 µg/mL for Zn^{2+}-ShPPase, 0.17 mg/mL for Zn^{2+}-$ShPPase^{D14N}$, and 0.12 mg/mL for Zn^{2}-$ShPPase^{D14A}$.

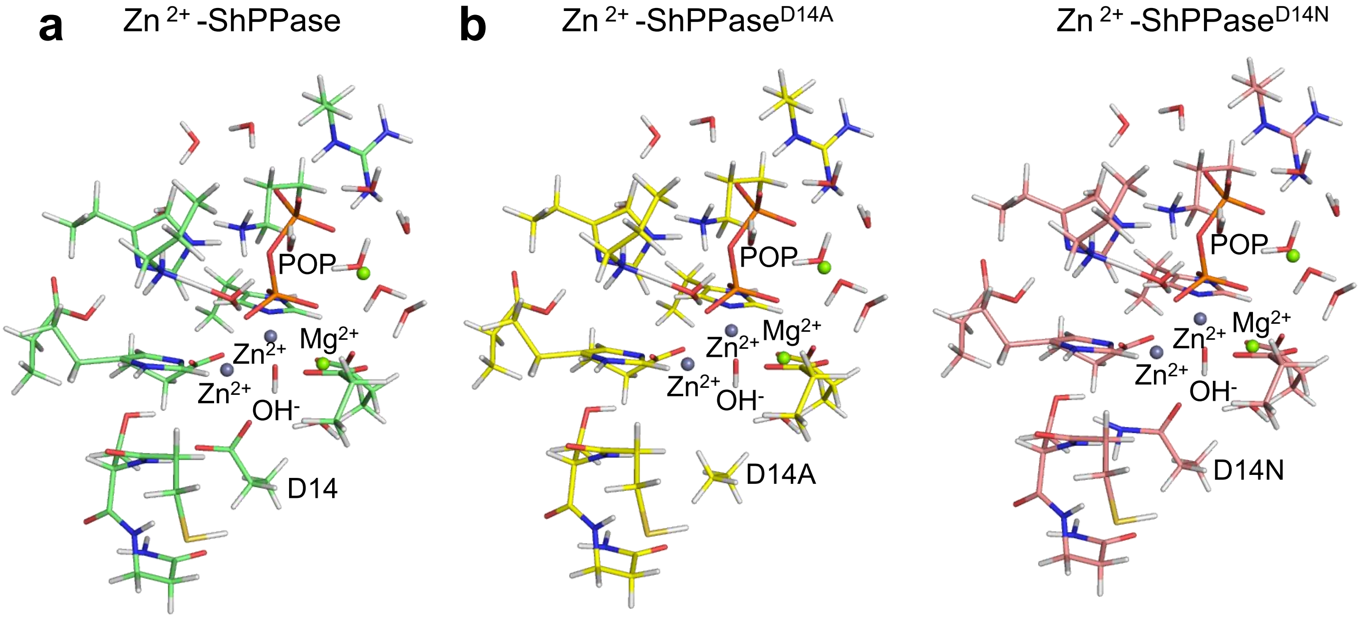

Supplementary Figure 9 | Comparison of optimized structures of (a) WT (light green) and (b) Asp14 mutation models for Zn-ShPPase^{D14A} (yellow) and Zn-ShPPase^{D14N} (pink). The Zn, Mg, O, N, P, and H atoms are colored slate purple, green, red, blue, orange, and white, respectively.

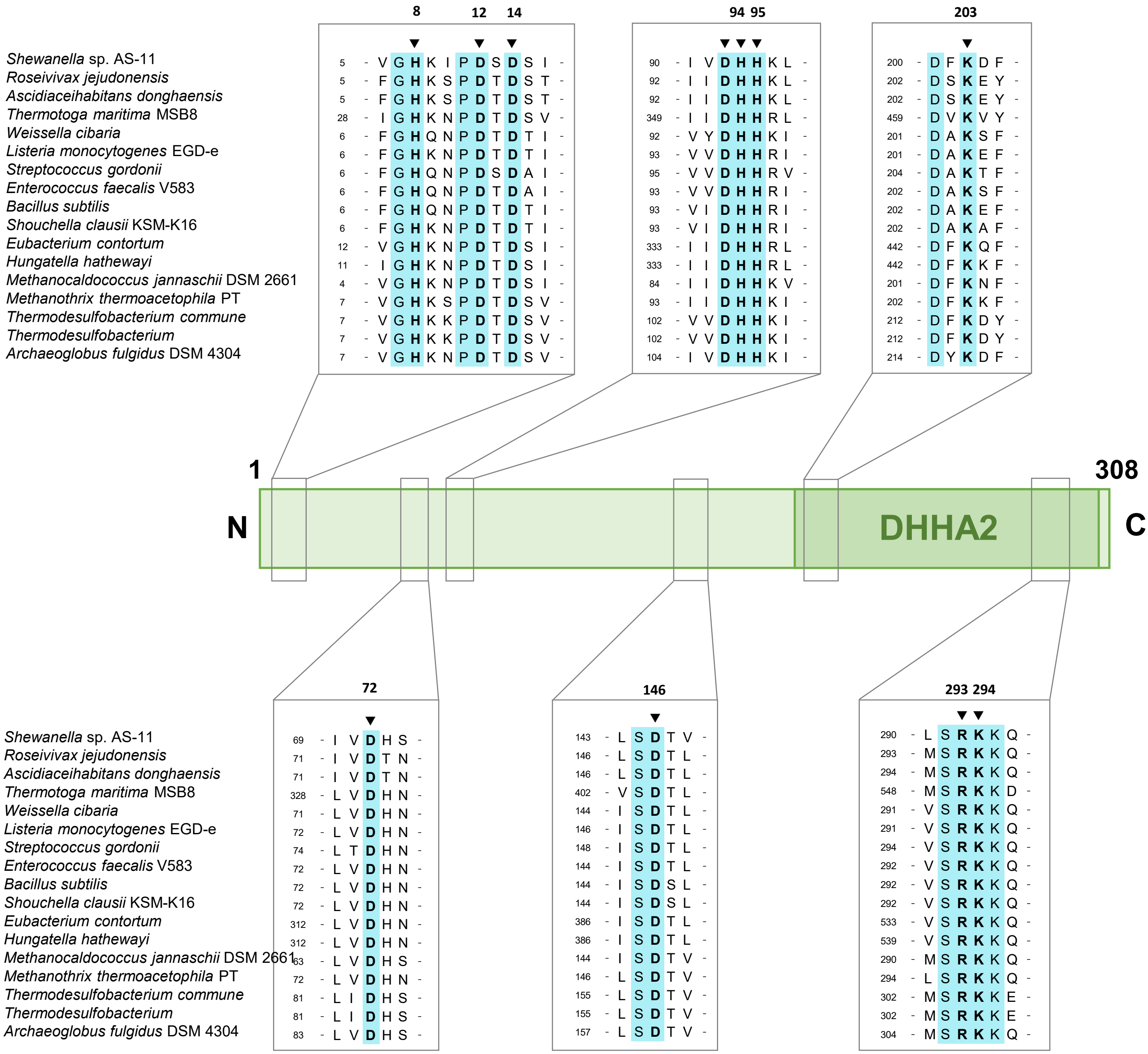

Supplementary Figure 10 | Multiple sequence alignments in reported Family II PPases. Amino acids of the active site are conserved among family II PPases and are shown in cyan (UniplotID: BAM74412.2, WP_085790359.1, SPH20124.1, AAD35672.1, KIU19850.1, CAC99526.1, AAB39104.1, AAO81394.1, BAA05186.1, BAD65437.1, CUO74905.1, CUO93176.1, AAB98601.1, ABK14034.1, HAA84199.1, WP_038063393.1, AAB90480.1). Asp 14 of ShPPase is completely conserved with others, which is shown in bold.

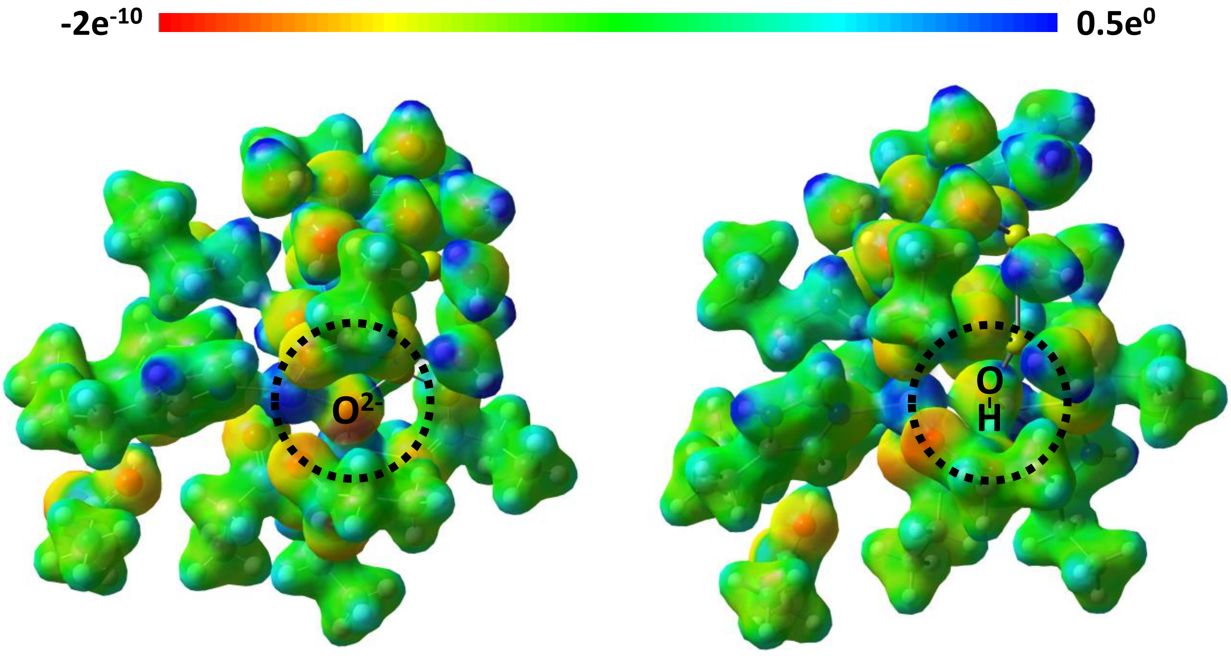

Supplementary Figure 11 | Electrostatic potential (ESP) map for IM2 state (left) and IM1 state (right). ESP of the ions of interest mapped onto the molecular surfaces with an electronic density of 0.001 au. Gray, white, blue, red, and orange represent C, H, N, O, and P atoms, respectively.

Supplementary Table 1 | Specific activity of sample for XRF

	Specific activity (U/mg)
Zn^{2+}-ShPPase	66 ± 13
Zn^{2+}-ShPPase for XRF	31 ± 6

The metal content of Zn2+-ShPPase was calculated based on the calibration curve (y=185.5x+59.418) with Zn standard solution (Supplementary Figure 8). The results indicated that the amount of zinc bound to the enzyme in the Zn^{2+}-ShPPase sample solution was 1.3 per molecule of enzyme. In addition, only the X-ray fluorescence spectrum of zinc was detected in the sample, indicating that only zinc, and not other non-target metals such as iron ions, is present in the active site. Furthermore, the activity of the Zn^{2+}-ShPPase sample for XAFS measurements and the fully activated Zn^{2+}-ShPPase sample were measured and compared. The fully activated Zn^{2+}-ShPPase sample was diluted in Activation buffer to be 0.5 mg/mL Zn^{2+}-ShPPase sample for XAFS measurement and left on ice for 2 hours to fully activate. As a result, the specific activity of the sample for XAFS measurement was 47% of that of the complete binuclear sample. Thus, 47% of the measured samples are binuclear, zinc ion-bound enzymes. Furthermore, since 1.3 zinc ions were bound per molecule, 36% were calculated to be uninuclear, and 17% to be without zinc ions. Therefore, it was determined that there were no free zinc ions in the sample solution that were not bound to the enzyme, which would be problematic for EXAFS analysis, due to the process of removing excess amounts of metal (ultrafiltration and spin columns).

Supplementary Table 2 | Damage caused by X-ray irradiation

	Specific activity (U/mg)
Zn^{2+}-ShPPase 1 scan	45 ± 3
Zn^{2+}-ShPPase 10 scan	31 ± 4

Values represent the mean ± s.d. from n=3 independent experiments.

Supplementary Table 3 | Comparison of fit statistics for different Zn coordination environments and constraint models

Type	Model	Chi-square	Reduced chi-square	R-factor	Interatomic distance (Å)				
					Zn-O	Zn-O (μ_3)	Zn-N	Zn-P	Zn-Zn
Coordination Test	O=3 N=1	102.12	16.28	0.053	2.03	-	2.17	-	-
	O=4 N=1	103.05	16.43	0.054	2.04	-	2.17	-	-
	O=5 N=1	104.17	16.61	0.054	2.04	-	2.17	-	-
	O=5 N=1 P=1 Zn=1	92.41	21.63	0.048	2.03	-	2.14	3.29	3.35
	O=4 O(μ_3)=1 N=1 P=1 Zn=1	67.05	20.49	0.035	2.02	1.82	2.14	3.31	3.33
Sensitivity Test	O=4 O(μ_3)=1 N=1 P=1 Zn=1	89.24	20.89	0.047	2.04	2.00	2.14	3.27	3.35
