# Supplementary material for: μ₃-Oxo nucleophile formation enables efficient SN2 hydrolysis at the trinuclear metal center in inorganic pyrophosphatase
Source: Commun Chem. 2026 Apr 2;9:190. doi: 10.1038/s42004-026-01996-7 (PMC13219714; doi:10.1038/s42004-026-01996-7)
Supplement: Supplementary file 3 — Description of Additional Supplementary Files [file 42004_2026_1996_MOESM3_ESM.pdf]

Description of Additional Supplementary Files:

File: Supplementary Data 1

Description: Atomic coordinates for optimized computational models.

This file contains the Cartesian coordinates (.xyz format) for all stationary points (reactants, intermediates, and transition states) involved in the catalytic mechanism of ShPPase discussed in the main text.

File: Supplementary Data 2

Description: The numerical source data underlying the graphs in the main figures (Figures 2, 3 and 5)

File: Supplementary Movie 1

Description: Formation of the hydrogen-bonding interaction between Asp14 and the μ_3-hydroxide via Asp14 rotation.

The imaginary-frequency vibrational mode of TS1, illustrating Asp14 rotation and concomitant hydrogen-bond formation with the μ_3- hydroxide. Blinking dashed lines indicate the original hydrogen bonds between Oδ2 of Asp14 and the Oγ and N atoms of Ser116 in the reactant state, as well as the newly formed hydrogen bond between Oδ2 and the μ_3-hydroxide in the IM1 intermediate. Zn, Mg, O, N, P, and H atoms are colored slate purple, green, red, blue, orange, and white, respectively.

File: Supplementary Movie 2

Description: Proton transfer and reorientation of protonated Asp14 toward Asp72 (rate-limiting step).

The imaginary frequency vibrational mode of TS2, illustrating proton transfer from the μ_3-hydroxide to Asp14 and concomitant reorientation of protonated Asp14 toward Asp72. Blinking dashed lines represent transient hydrogen bonding interactions between Oδ2 of protonated Asp14 and the μ_3-oxo group, as well as Oδ2 of Asp72. Zn, Mg, O, N, P, and H atoms are colored slate purple, green, red, blue, orange, and white, respectively.

File: Supplementary Movie 3

Description: SN2-type hydrolysis mediated by the μ_3-oxo nucleophile.

The imaginary-frequency vibrational mode of TS3, illustrating nucleophilic attack of the μ_3-oxo species at the phosphorus center in an SN2-type transition state. Zn, Mg, O, N, P, and H atoms are colored slate purple, green, red, blue, orange, and white, respectively.
