# Supplementary material for: μ₃-Oxo nucleophile formation enables efficient SN2 hydrolysis at the trinuclear metal center in inorganic pyrophosphatase
Source: Commun Chem. 2026 Apr 2;9:190. doi: 10.1038/s42004-026-01996-7 (PMC13219714; doi:10.1038/s42004-026-01996-7)
Supplement: Supplementary file 9 — Reporting Summary [file 42004_2026_1996_MOESM9_ESM.pdf]

nature portfolio

Corresponding author(s): Keiichi Watanabe

Last updated by author(s): Mar 13, 2026

Reporting Summary

Nature Portfolio wishes to improve the reproducibility of the work that we publish. This form provides structure for consistency and transparency in reporting. For further information on Nature Portfolio policies, see our Editorial Policies and the Editorial Policy Checklist.

Statistics

For all statistical analyses, confirm that the following items are present in the figure legend, table legend, main text, or Methods section.

n/a	Confirmed	
☐	☒	The exact sample size (*n*) for each experimental group/condition, given as a discrete number and unit of measurement
☐	☒	A statement on whether measurements were taken from distinct samples or whether the same sample was measured repeatedly
☒	☐	The statistical test(s) used AND whether they are one- or two-sided<br>*Only common tests should be described solely by name; describe more complex techniques in the Methods section.*
☒	☐	A description of all covariates tested
☒	☐	A description of any assumptions or corrections, such as tests of normality and adjustment for multiple comparisons
☐	☒	A full description of the statistical parameters including central tendency (e.g. means) or other basic estimates (e.g. regression coefficient) AND variation (e.g. standard deviation) or associated estimates of uncertainty (e.g. confidence intervals)
☒	☐	For null hypothesis testing, the test statistic (e.g. *F*, *t*, *r*) with confidence intervals, effect sizes, degrees of freedom and *P* value noted<br>*Give P values as exact values whenever suitable.*
☒	☐	For Bayesian analysis, information on the choice of priors and Markov chain Monte Carlo settings
☒	☐	For hierarchical and complex designs, identification of the appropriate level for tests and full reporting of outcomes
☒	☐	Estimates of effect sizes (e.g. Cohen's *d*, Pearson's *r*), indicating how they were calculated

Our web collection on statistics for biologists contains articles on many of the points above.

Software and code

Policy information about availability of computer code

Data collection	X-ray absorption spectroscopy data were collected at beamline BL15 of the SAGA Light Source using the beamline data acquisition system associated with the synchrotron instrumentation. XAFS spectra at the Zn K-edge were recorded using a Si(111) double-crystal monochromator and detected as Zn Kα fluorescence using a 7-element silicon drift detector (Techno-AP). Data acquisition was performed using the standard beamline control software provided by the facility. No custom computer code was developed or used for data collection.
Data analysis	XAFS data processing and analysis were carried out using the Demeter software package (version 0.9.26), including the programs Athena and Artemis. Individual scans were normalized to the incident photon flux and averaged using Athena. Background subtraction, normalization, and spectral processing were performed following standard XAFS protocols. EXAFS fitting was performed using Artemis with theoretical scattering paths generated by FEFF version 7.0.Quantum mechanical calculations were performed using density functional theory (DFT) implemented in the Gaussian 16 program package. Geometry optimizations and vibrational frequency analyses were carried out using the B3LYP functional with appropriate basis sets for each atom type. Protonation states were evaluated using the Amber force field implemented in the Molecular Operating Environment software. Visualization and animation of molecular structures and vibrational modes were performed using Chemcraft (version 1.8).No custom computer code was developed for the data analysis in this study.

For manuscripts utilizing custom algorithms or software that are central to the research but not yet described in published literature, software must be made available to editors and reviewers. We strongly encourage code deposition in a community repository (e.g. GitHub). See the Nature Portfolio guidelines for submitting code & software for further information.

Data

Policy information about availability of data

All manuscripts must include a data availability statement. This statement should provide the following information, where applicable:

- Accession codes, unique identifiers, or web links for publicly available datasets
- A description of any restrictions on data availability
- For clinical datasets or third party data, please ensure that the statement adheres to our policy

The data that support the findings of this study are available within the paper and its Supplementary Information files. The atomic coordinates for the optimized computational models are provided as Supplementary Data 1. Source data underlying the figures and tables are provided as Supplementary Data 2. The protein crystal structures analyzed in this study are available from the Protein Data Bank under accession codes 6LL7 and 6LL8. All other raw data, including X-ray absorption spectroscopy and enzyme activity assay data, are available from the corresponding author upon reasonable request.

Research involving human participants, their data, or biological material

Policy information about studies with human participants or human data. See also policy information about sex, gender (identity/presentation), and sexual orientation and race, ethnicity and racism.

Reporting on sex and gender	No human participants or human data were involved in this study; therefore sex and gender were not considered.
Reporting on race, ethnicity, or other socially relevant groupings	No human participants or human data were involved in this study; therefore race, ethnicity, or other socially relevant groupings were not applicable.
Population characteristics	No human participants were included in this study.
Recruitment	No human participants were recruited for this study.
Ethics oversight	Ethics approval was not required because this study did not involve human participants, human data, or human biological materials.

Note that full information on the approval of the study protocol must also be provided in the manuscript.

Field-specific reporting

Please select the one below that is the best fit for your research. If you are not sure, read the appropriate sections before making your selection.

☒ Life sciences ☐ Behavioural & social sciences ☐ Ecological, evolutionary & environmental sciences

For a reference copy of the document with all sections, see nature.com/documents/nr-reporting-summary-flat.pdf

Life sciences study design

All studies must disclose on these points even when the disclosure is negative.

Sample size	For enzyme activity measurements, kinetic parameters (Km and kcat) were determined from three independent measurements and are reported as mean ± s.d. For X-ray absorption spectroscopy (XAFS), each spectrum represents the average of 10 scans collected for each sample to improve the signal-to-noise ratio. DFT calculations were performed on defined computational models; therefore no statistical sample size determination was required.
Data exclusions	No data were excluded from the analyses. All measurements and computational results obtained during the study were included in the reported analyses.
Replication	Enzyme activity measurements were repeated independently three times to ensure reproducibility. For XAFS measurements, multiple scans (10 scans per sample) were collected and averaged to improve data quality and reproducibility. Computational analyses were performed using established quantum chemical methods.
Randomization	Randomization was not applicable to this study because no experimental groups or treatments requiring random allocation were used.
Blinding	Blinding was not applicable to this study because the experiments did not involve subjective assessment or group allocation.

Reporting for specific materials, systems and methods

We require information from authors about some types of materials, experimental systems and methods used in many studies. Here, indicate whether each material, system or method listed is relevant to your study. If you are not sure if a list item applies to your research, read the appropriate section before selecting a response.

Materials & experimental systems

n/a	Involved in the study
☒	☐ Antibodies
☒	☐ Eukaryotic cell lines
☒	☐ Palaeontology and archaeology
☒	☐ Animals and other organisms
☒	☐ Clinical data
☒	☐ Dual use research of concern
☒	☐ Plants

Methods

n/a	Involved in the study
☒	☐ ChIP-seq
☒	☐ Flow cytometry
☒	☐ MRI-based neuroimaging

Plants

Seed stocks	No plants were used in this study.
Novel plant genotypes	No plant materials or plant genotypes were used in this study.
Authentication	Not applicable because no plant materials were used in this study.
